# The Effect of Olfactory Exposure to Non-Insecticidal Agrochemicals on Bumblebee Foraging Behavior

**DOI:** 10.1371/journal.pone.0076273

**Published:** 2013-10-24

**Authors:** Jordanna D. H. Sprayberry, Kaitlin A. Ritter, Jeffrey A. Riffell

**Affiliations:** 1 Department of Biology, Muhlenberg College, Allentown, Pennsylvania, United States of America; 2 Department of Biology, University of Washington, Seattle, Washington, United States of America; University of California, Los Angeles, United States of America

## Abstract

Declines in bumblebee populations have led to investigations into potential causes – including agrochemical effects on bumblebee physiology. The indirect effects of agrochemicals (i.e. behavior modulation) have been postulated, but rarely directly tested. Olfactory information is critical in mediating bumblebee-floral interactions. As agrochemicals emit volatiles, they may indirectly modify foraging behavior. We tested the effects of olfactory contamination of floral odor by agrochemical scent on foraging activity of *Bombus impatiens* using two behavioral paradigms: localization of food within a maze and forced-choice preference. The presence of a fungicide decreased bumblebees’ ability to locate food within a maze. Additionally, bumblebees preferred to forage in non-contaminated feeding chambers when offered a choice between control and either fertilizer- or fungicide-scented chambers.

## Introduction

Bumblebees are important pollinators in both natural and agricultural ecosystems[1-3]. Unfortunately bumblebees have been experiencing alarming declines in recent decades [4-6]. Multiple factors appear to be contributing to bumblebee declines; including reduction in native habitat [7], habitat fragmentation [7,8], pesticide exposure [4,7,9], and pathogens [4,5]. Bumblebees may be particularly sensitive to the effects of agrochemicals as previous work has shown that only the largest colonies successfully produce queens [10,11]. Given that foraging workers are responsible for provisioning the hive [12], interference with foraging behavior could impact reproductive success. Indeed, sublethal exposure to neonicotinoid pesticides significantly decreased the weight of field-foraging bumblebee colonies and drastically reduced queen production [9]. Moreover, Gill et al linked pesticide-induced reduction in forager efficiency with lower colony fitness [13]. 

The lethal and sublethal effects of exposure to neonicotinoid pesticides on bees have been addressed in numerous studies (reviewed by Blacquiere et al [14]). The physiological and behavioral effects of other agrochemicals, such as fungicides, are less clear. As fungicide residues have been found alongside pesticides in honeybee colonies [15], they are likely present in bumblebee colonies as well. Fungicides have interactive effects - increasing the toxicity of some pesticides [16,17]. Demethylation inhibitor (DMI) fungicides have been found to adversely affect larval and pupal development in cabbage moths [18], as they are potent inhibitors of ecdysteroid and juvenile hormone biosynthesis[19,20]. Our understanding of fungicide effects on bee behavior is limited to the disruptive effects of azole fungicide exposure on honeybee thermogenesis, an effect that is exacerbated by coexposure to a pyrethroid insecticide [21].

In addition to the potential direct effects of agrochemicals on bumblebees, there is the possibility of indirect effects on bumblebee health through modification of foraging patterns. The reduction of forage availability through chemically enhanced agriculture has been explored [4,22], but there could also be effects via alteration of floral sensory signals. Flowers exhibit a variety of traits that serve as attractive ‘signals’ to pollinators, and while visual signals are critical in resource localization [23-25] flowers also have morphological [26] and olfactory components [27]. Floral odorants are attractive to pollinators [28,29], and have been shown to mediate bumblebee-flower interactions [30,31]. Work on floral signal-complexity indicates that scent improves both learning [30] and visitation rates [31] in bumblebees. Given the importance of olfactory signals in pollinator foraging behavior, how pollutants, including agrochemicals, interfere with floral odorants could be of critical importance. Although the effects of air pollutants (such as ozone) on floral signal fidelity have been studied [32,33], the behavioral impacts of olfactory contamination remain unexplored. To begin to address these gaps, the aim of our study was to determine whether or not the scent of common agrochemicals interfered with foraging behavior in bumblebees.

## Materials and Methods

**Figure 1:**
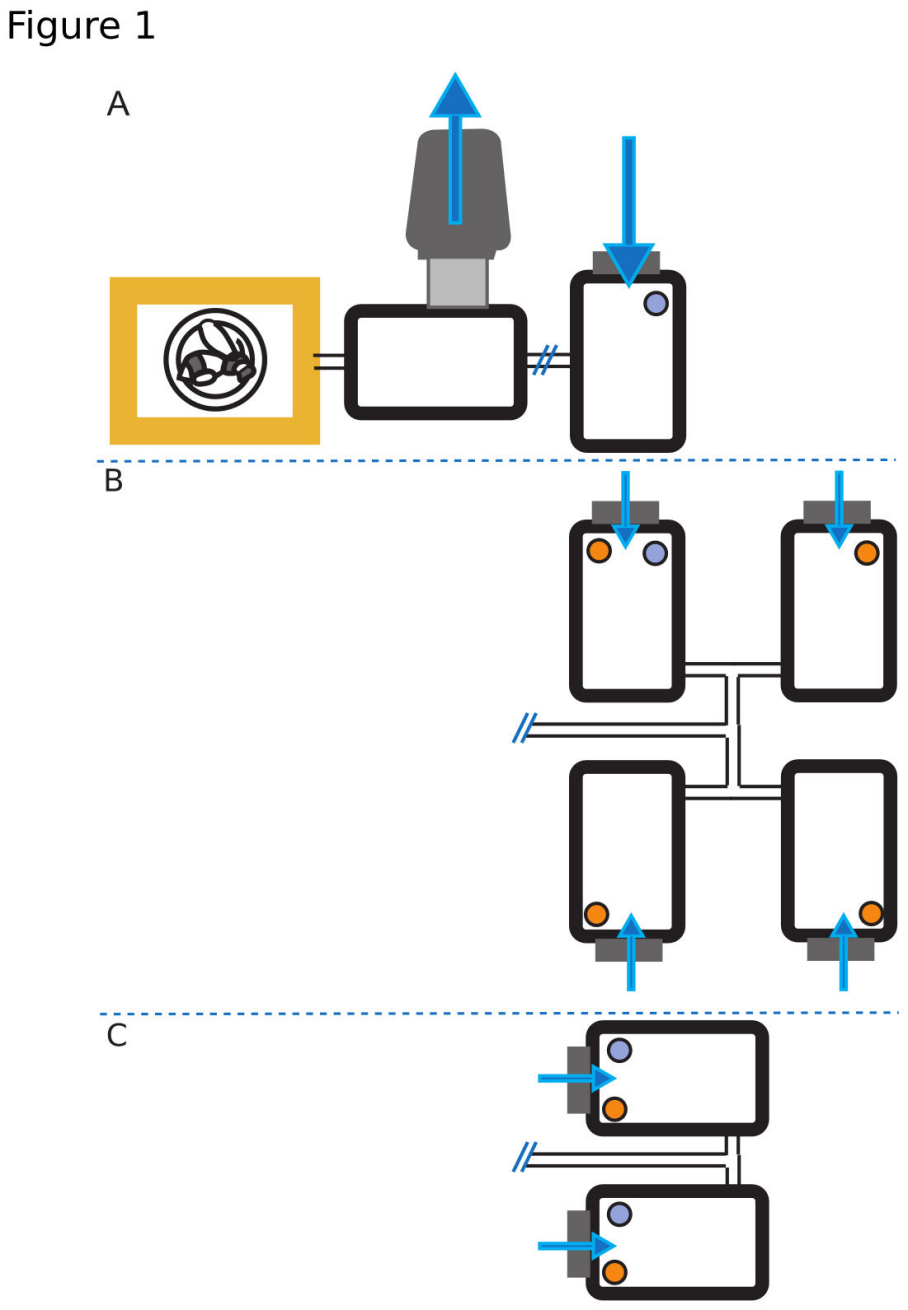
Experimental set-up of (BioBest) *Bombus impatiens* hives. (a) During the training phase and on non-testing days, bumblebees were given access to a single foraging chamber containing a linalool-scented feeder (represented by the purple circle) for 2-3 hours per day. A 4” duct fan pulled air through the foraging chamber and vented it through a large activated-charcoal filter to prevent odor contamination of the lab space. The air-inlet to the foraging chamber was also fitted with an activated charcoal filter. Arrows indicate direction of airflow. (b) For maze trials, the single feeding chamber was replaced with a double-T maze. The maze was either unpolluted, Turf Builder-permeated, or Manzate permeated. Each chamber was treated with an appropriate amount of chemical (3.9 g Turf Builder and 0.09 g Manzate), as indicated by the orange circles. A single linalool feeder was randomly placed in a single chamber of the maze. (c). For preference experiments, the single feeding-chamber was replaced with 2 chambers in a T configuration. Each chamber contained a linalool scented feeder and a ‘pollutant’ jar. In control trials, both pollutant jars were empty. In agrochemical-trails, one randomly selected chamber’s jar contained either Turf Builder (3.9 g) or Manzate (0.09 g).

Behavioral investigations (a total of 111 trials) utilized seven commercial (BioBest) *Bombus impatiens* colonies. Colonies of *Bombus impatiens* were maintained in the lab with a 16h/8h light/dark cycle (the 16 hours of light were subdivided into 2h dawn / 12h day/ 2h dusk). Bumblebees had ad libitum access to pollen in an antechamber attached to the hive. Colonies were given access to a glass feeder with 66% BeeHappy (a proprietary nectar blend provided by BioBest) in an attached foraging chamber for a limited time window (2-3 hrs) each day. Filter paper with 15 uL of a 1:1000 dilution of linalool (a monoterpene VOC [ > 95% purity, Sigma Co.]) was placed on top of the feeder. An exhaust fan situated in the antechamber was turned on to draw air from the foraging chamber into the antechamber. Both the exhaust outlet and the air intake (in the foraging chamber) were fitted with activated charcoal filters to prevent unintended odorant transfer (Figure 1a). 

 Two different behavioral assays were performed. The first used a maze assay to determine if agrochemicals modified foraging performance, while the second used a T-configuration to explicitly test whether agrochemicals affected bumblebee olfactory preferences. Agrochemical treatments were consistent throughout: Turf Builder (Scott’s®), a common lawn fertilizer & herbicide, and Manzate (DuPont®), a fungicide typically used in larger scale agricultural applications. Both of these chemicals were tested to confirm that they indeed off-gassed volatile compounds (i.e. had a ‘scent’), and for interactions with linalool in the head space to form novel compounds (see *headspace sampling of volatiles*).

### Maze Experiments

#### Training

Individual bees on the linalool-scented feeder were tagged (http://www.thorne.co.uk) and tracked. A tagged bee was considered ‘trained’ if we recorded three visits to the feeder within five days. 

#### Experimental Protocol

The single training chamber was replaced with a four-chamber double-T maze for experimental trials (Figure 1b). The four chambers comprising the maze were identical to the training chamber. Each chamber was placed the same distance from the entrance resulting in an average flow rate of 0.26 +- 0.02 m/s through the maze (measured at the entrance to each chamber with a VWR Traceable Hot Wire Anemometer, model #21800-024). Trained bees were randomly selected to run one of three different maze treatments: control, Turf-Builder, or Manzate. For control trials each chamber received an empty screen-topped glass jar and a single (randomized) chamber received a linalool-scented feeder. For pollutant trials, each chamber’s glass jars contained the manufacturer’s recommended application based upon the square footage of the maze’s footprint (12 ft^2^) divided into four chambers. Turf builder was applied at a rate of 3.4 g/chamber and Manzate was applied at a rate of 0. 9 g/chamber. As per manufacturer’s instructions, the turf builder was lightly moistened. As with the control trials, a randomized chamber received a linalool-scented feeder. Based upon feeder placement, bumblebees would need to enter the chamber to confirm feeder presence. Given the absence of visual cues, bumblebees should be predominately using olfactory cues to locate feeders within the maze.

Trained bumblebees were released into the maze individually. We recorded the first chamber they entered (“first chamber choice”) and the time it took to reach a feeder. Bees that did not locate the feeder after 15 minutes were returned to the hive and excluded from analysis (13 of 51 maze runs by 35 bees from 4 colonies). After each trial the maze was removed, swabbed with alcohol to remove any scent marks or residual odors, and dried in a hood. The plastic chambers for control and pollution mazes were maintained separately, to prevent any potential odor absorption and re-emission from confounding the results. 

#### Analysis

To avoid corruption of the results from learning effects, we only analyzed bumblebees’ first runs through the maze (26 individuals out of 51 trials). The two primary experimental results recorded from these experiments were choice accuracy and time to feeder. Choice accuracy was calculated as the percentage of all bumblebees within a treatment that chose the chamber containing the feeder on their first try (‘choice’ being defined as a bee entering a chamber). While percentage data might typically analyzed with a Pearson’s Chi Square analysis, due to lower sample sizes we used a Binomial exact test. To determine whether or not bumblebees were performing better than chance, choice data were run against an expected success rate of 25%. To determine whether or not chemical treatments negatively impacted choice performance, the Turf Builder and Manzate data were analyzed using the control performance (89%) as the expected success rate, and the p values were Bonferroni corrected to account for multiple comparisons. Time to feeder was analyzed using an ANOVA and a Tukey’s HSD post-hoc test to elucidate which comparisons were significant. As timing data could be confounded by incorrect choices (i.e. bumblebees that chose the wrong feeding chamber on their first try will most likely have a longer time to feeder), we repeated the timing-analysis using only those trials in which bumblebees made accurate first-chamber choices. We also used an ANOVA to confirm that both the colony of origin and the chamber location did not have significant impacts (table 1). The statistics for these and all other analyses were done in the open source software package R (http://www.r-project.org).

**Table 1:** 

Variable 1	Vs. Variable 2	F	P
Time to feeder	Pollution treatment	5.13	0.014
Time to feeder (*excluding incorrect choices*)	Pollution treatment	18.262	<0.001
Time to feeder	Hive	0.827	0.493
Time to feeder	Chamber	0.0717	0.791

An ANOVA was used to determine which factors determined changes in time to feeder. The only significant factor was pollution treatment; the bees’ hive of origin and the chamber location of the feeder had no effect.

### Preference Experiments

#### Training

The training paradigm for these experiments was similar to maze experiments, with one exception. Since individual bees were not tracked, the colony at large had exposure to a linalool-scented feeder for a minimum of five consecutive days prior to participating in preference trials.

#### Experimental Protocol

As with maze experiments, preference trials fell into one of three treatment groups: control, Turf-Builder, or Manzate. In these experiments, the single feeding chamber was replaced with two chambers in a T configuration (Figure 1c). Each chamber was equipped with a linalool-scented feeder. In control trials each chamber held an empty glass jar. In agrochemical trials, one chamber held an empty jar, while the other chamber had a jar containing the relevant agrochemical (3.9 g Turf Builder, 0.09 g Manzate). Fifteen minutes after bumblebees were granted access to the foraging chambers the number of bumblebees on the feeder in each chamber was counted. This count was repeated every fifteen minutes until one hour had passed. The location of the polluted chamber was randomized, and chamber location did not have an effect on preference (Wilcoxon signed-rank test: Z=-1.354, p = 0.178 for 15-minute data; Z=0.005, p=0.998 for 60-minute data).

#### Analysis

Given high variability in the number of foraging bees between days and colonies, these experiments were designed to use a pair-wise statistical analysis to compare relative distributions of bumblebees between treatment chambers on an individual day. To prevent intercolony variation from skewing the data, treatments were distributed across the three colonies used in these experiments (table 2). As the distribution of treatments across colonies was not perfectly balanced, a repeated-measures ANOVA was not preferable. Data from individual trials were included if the average number of bees in either chamber (at 60 minutes) exceeded 2/3 average number of bees in the least populated chamber on the first control trial for a given colony (48 of 63 trials). Given the non-normality of some data sets, this analysis utilized a Wilcoxon rank sum test (corrected for ties to allow calculation of exact p-values) rather than a paired t-test.

**Table 2:** 

**Colony**	**Control**	**Turf Builder**	**Manzate**
**A**	9	7	8
**B**	4	4	1
**C**	3	6	6

The distribution of trials across colonies in preference experiments**.**

### Headspace sampling of volatiles and gas chromatography with mass spectrometric (GCMS) detection

To analyze the volatiles that the Turf Builder and Manzate emitted, as well as to determine if those emissions influenced our odorant stimuli, we used solid phase microextraction fibers (SPME) exposed to the headspace of the different treatments. 100-mg of either Manzate of Turf Builder was added to a 10-mL headspace sample vial with associated septa (Agilent Technologies, Palo Alto, CA, USA). 100-mg of Manzate or Turf Builder was also added to ample vials containing 1-ug of (±)-linalool (99% purity, Sigma Co.). Vials were incubated at 23 °C for 30 min, after which time the SPME Fiber (57344-U, 75 μm Carboxen-PDMS; Supelco Analytical, Bellafonte PA USA) was inserted into the vial and exposed for 30 min. Volatiles absorbed on the SPME fiber were then analyzed using GC- with mass spectrometric detection (GC-MS) consisting of an HP 7890A GC and a 5975C Network Mass Selective Detector (Agilent Technologies, Palo Alto, CA, USA), with the inlet temperature set to 220 °C to volatilize the absorbed compounds on the fiber. A DB5 GC column (J&W Scientific, Folsom, CA, USA; 30m, 0.25mm, 0.25um) was used, and helium was used as carrier gas at constant flow of 1cc/min. The initial oven temperature was 50 C for 5 min, followed by a heating gradient of 10 C/min to 350C, which was held isothermally for 10 min. Chromatogram peaks were identified tentatively with the aid of the NIST mass spectral library (ca, 120,000 spectra), verified by chromatography with authentic standards (when available) and published Kovats Indices. Peak areas for each compound were integrated using Chem Station software (Agilent Technologies, Palo Alto, CA, USA) and are presented in nanograms. Four to nine replicates were conducted for each treatment group. 

## Results

### Maze Experiments

**Figure 2:**
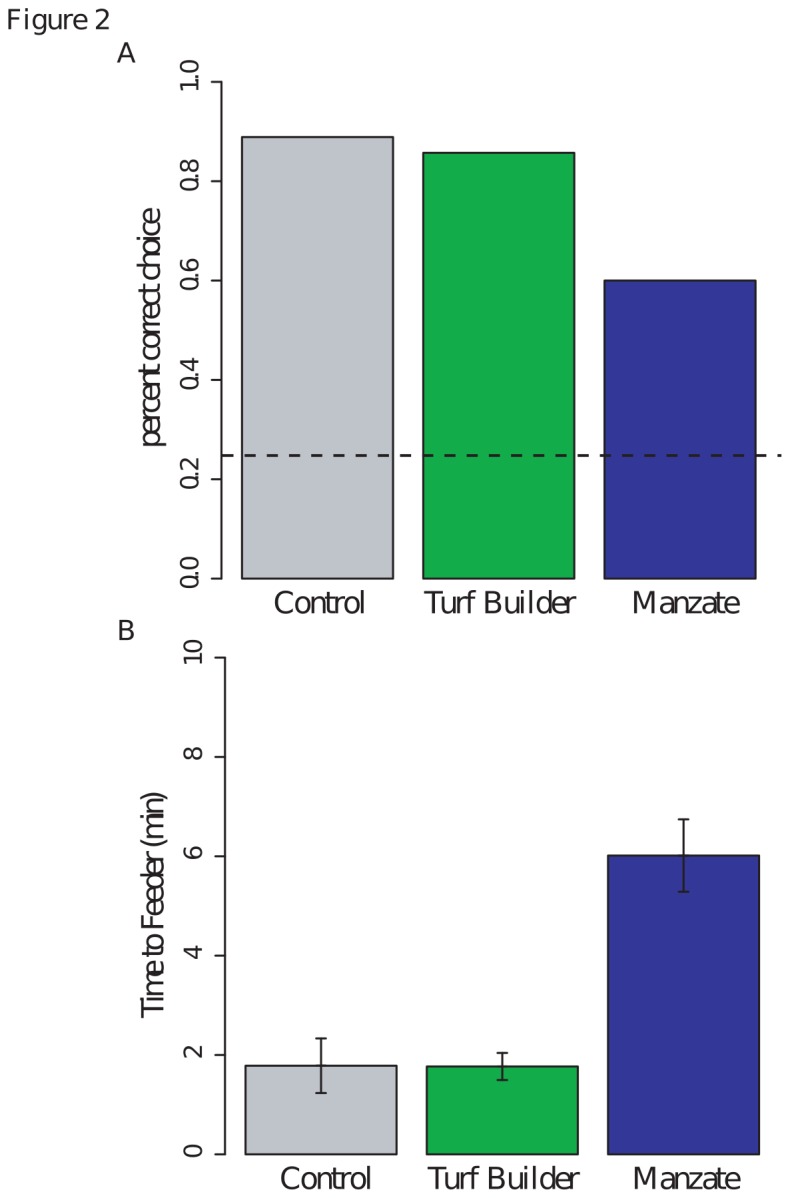
Bumblebees running through Manzate-permeated mazes showed decreases in measured performance metrics. (a) The percentage of bumblebees within a treatment group that successfully located the feeder within the maze on their first try (success due to random chance is represented by the dashed line). All three treatment-groups performed significantly better chance [Binomial exact test, p=0.0001 (control, n=9), p=0.001 (Turf Builder, n=7), p=0.02 (Manzate, n=10)], but only the Manzate treatment group showed a significant decrease in performance when compared to control [Binomial exact test, p=0. 0356 (Manzate), p=1.0 (Turf Builder)]. (b) Bumblebees running through Manzate-permeated mazes took a significantly longer time to locate the feeders [ANOVA, Tukey’s HSD, p<0.001]. These data exclude incorrect choice events.

Bumblebees running through unpolluted and Turf Builder-permeated mazes located the feeder with high accuracy (>85%, Figure 2a; Binomial exact test: p=0.0001, n=9 and 0.001, n=7 respectively). Interestingly, while bumblebees running a Manzate-permeated maze perform significantly better than chance (60% accuracy versus 25%; p=0.02, n=10), they made incorrect choices more frequently than bumblebees running through an unpolluted maze (*Bonferroni corrected* p=0.0356) – an effect not demonstrated in bumblebees running Turf Builder-permeated mazes (*Bonferroni corrected* p=1.0). 

In addition to decreasing choice accuracy, Manzate significantly increased the time it took for bumblebees to locate the feeder (Figure 2b; ANOVA & Tukey’s HSD: p=0.02). We still see this increase if we exclude bees that made incorrect choices, thereby controlling for those bees that might become “lost” in the maze (p<0.001). The brief exposure to Manzate or Turf Builder did not appear to have obvious long-term physiological effects; after testing, bumblebees’ likelihood of returning to the maze was independent of their chemical exposure (table 3; Fisher’s exact test: p=0.79, n=34).

**Table 3:** 

Exposure	Return	Non-return
Control	3	10
TB	2	7
Manzate	4	8

While the final analysis only included bumblebee’s first (naïve) run through the maze, many bumblebees did in fact run through the maze multiple times. The return column indicates the number of times a bee returned to re-run the maze after being exposed to a particular chemical. The non-return column shows the number of times a bee did not return to re-run the maze after being exposed to a particular chemical. A Fisher’s exact test indicates that prior chemical exposure has no effect on the likelihood of a bee to return to the maze (p=0.79).

### Preference Experiments

**Figure 3:**
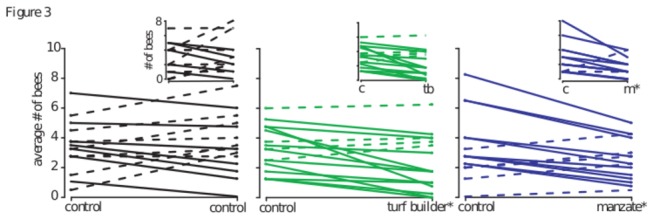
Bumblebees preferred to feed in unpolluted environments. Data depicted compare the number of bees on the feeder in a control (unpolluted) chamber and a polluted chamber (Turf Builder or Manzate-permeated). Solid lines indicate decreasing trends (more bees in the control chamber), while dashed lines indicate increasing trends. The full size graphs show 60-minute averages. Both Turf Builder and Manzate treatment groups show a significant preference for the unpolluted condition [Wilcoxon signed rank test: p=0. 6462 (control, n=15), p= 0. 0079 (Turf Builder, n=16), p= 0. 0154 (Manzate, n=15)]. The insets show the 15-minute count, when the Manzate group is already showing a significant preference [Wilcoxon signed rank test: p= 0. 8438 (control, n=16), p= 0. 1449 (Turf Builder, n=17), p= 0. 0083 (Manzate, n=15)]. *p<0.05.

At fifteen minutes bumblebees exhibited a significant preference for feeding in the unpolluted chamber versus the Manzate-permeated chamber (Wilcoxon signed-rank test: Z=2.5671, p=0.0083); this effect was maintained in the 60-minute average (Figure 3; Z=2.3618, p=0.0154). Interestingly, although bumblebees demonstrated an inconclusive lack of preference for the unpolluted- versus the Turf Builder-chamber at the 15-minute mark (Figure 3; Z=1.4819, p=0.1449), the 60-minute averages showed a significant effect (Z=2.5652, p=0.0079). As expected, control trials did not exhibit a preference for either unpolluted chamber (Figure 3; Z=-0.2113, p=0.8438 and Z=-0.4837, p=0.6462 for 15- and 60-minute measures respectively). These preference data confirm the negative impact that Manzate has on foraging activity in *B. impatiens*. Interestingly, the preference data imply that Turf Builder may interfere with olfactory preferences, despite having no impact on navigation performance.

### Headspace sampling of volatiles

**Figure 4:**
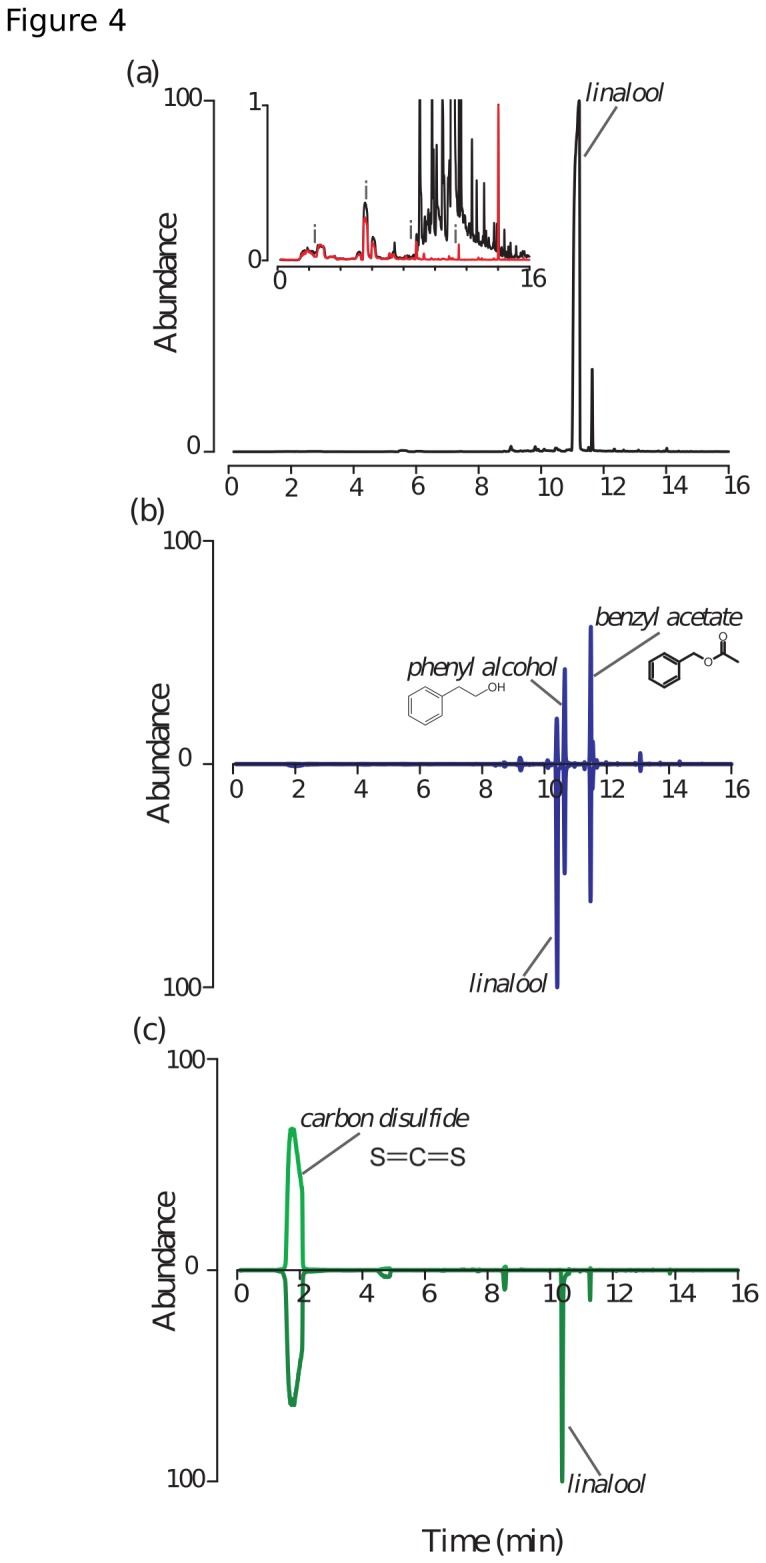
Total ion chromatograms of the different chemical treatments. (a) (±)-linalool (99% Sigma) spiked with BeeHappy – the zoomed inset shows BeeHappy alone (red line) overlain on the combined trace, (b) Turf Builder (top) and Turf Builder spiked with (±)-linalool (bottom), and (c) Manzate (top) and Manzate spiked with (±)-linalool (bottom).

Analysis of volatiles emitted from the BeeHappy nectar (see GCMS below) demonstrated no quantifiable levels of linalool or oxygenated monoterpenes (Figure 4a), thus indicating that the conditioned (linalool) and unconditioned (BeeHappy) stimuli are activating distinct sensory channels. Regardless, BeeHappy nectar was used in both training and testing; therefore olfactory cues remained constant in both phases.

Both agrochemicals tested did indeed off-gas volatile compounds. Interestingly, Turf Builder contained linalool, as well as some other flower volatiles (e.g., phenyethyl alcohol and benzyl acetate) (Figure 4b). By contrast, Manzate’s scent profile was less complex, predominantly off-gassing carbon disulfide (Figure 4c). The presence of the agrochemical-emitted volatiles did not affect the emissions of linalool in the headspace. The small difference in linalool retention time between the "BeeHappy" samples and the manzate and turf-builder reflect different hold times in the beginning of the runs.

## Discussion

The data presented here show modification of bumblebee behavior based upon exposure to agrochemical *scent alone*. To our knowledge, this study is the first to document such a behavioral effect, and provides strong impetus for several novel areas of investigation.

Bumblebees showed increased navigation time when exposed to the scent of Manzate, a commercial fungicide. As increased foraging time of bumblebee workers scales up to meaningful decrements in colony fitness [13], it is reasonable to hypothesize that consistent olfactory contamination by Manzate in the field could negatively impact both native and commercial bumblebees. However, the consistency of olfactory contamination is currently unknown. Manufacturer recommendation for application varies from 3 to 14 days, with many crops residing in the 7-10 day range. As we do not know how long Manzate off-gasses, understanding the volatile behavior of Manzate in the field is an interesting avenue for further investigation. Given the reduced preference for Manzate-contaminated feeding chambers, this information would also be relevant to understanding if and for how long farmers could expect lower visitation to treated crops.

Despite the fact that Turf Builder (a residential fertilizer and herbicide) did not have significant impacts on maze-navigation, our data indicate that bumblebees in the lab still actively preferred to feed in an uncontaminated environment. This could be due to an active aversion to the contaminating scent, or an artifact of bumblebees preferring a learned olfactory signal. Given this ambiguity, understanding how these data will translate to behavior of bumblebees in urban, suburban and agricultural settings will require field observations and experimentation. 

The observed effects of agrochemical scent-contamination of learned odors may be due to behavioral modification mediated via the olfactory system. This could be through modification of sensory signal processing, changes of olfactory blend structure resulting in changes to neural representation of olfactory signals by the antennal lobe [34], or modification of perception/ activity in higher order learning and integration centers such as the mushroom bodies [35]. Indeed, higher order learning centers in the honeybee have been the target of investigation and hypotheses about sublethal effects of pesticides [36]. However, these studies are considering physiological exposure via diet, giving ‘blanket’ effects on cholinergic and octopaminergic signaling. The route of exposure in our experiments implies effects routed through the olfactory sensory pathway, rather an impact on the whole nervous system. Preliminary measurements of antennal field potential during stimulation with agrochemicals further support this hypothesis (Sprayberry, unpub. data). An alternate, residual, explanation for the behavioral effects may be through volatile delivery to the body tissue by the tracheal respiratory system [37], thereby potentially modulating the nervous and/ or motor systems. However, the lack of significant effects of Turf Builder odor in maze experiments, but an active avoidance of it in preference trials implies involvement of sensory and learning pathways. Future investigations will seek to elucidate potential neural mechanisms for observed behavioral changes. 

Regardless of mechanism, the data presented here indicate that the indirect effects of agrochemicals result in meaningful behavioral changes in a critical pollinating insect. Given the pollination crisis our agricultural systems are facing, these indirect effects are worthy of consideration. 

## Supporting Information

Data S1 **The file Data S1 is an excel spreadsheet containing the raw data for all analyses presented in this manuscript.**
(XLSX)
